# Indium Nitrite (InN)-Based Ultrasensitive and Selective Ammonia Sensor Using an External Silicone Oil Filter for Medical Application

**DOI:** 10.3390/s18113887

**Published:** 2018-11-11

**Authors:** Sujeet Kumar Rai, Kun-Wei Kao, Shanjgr Gwo, Ashish Agarwal, Wei Da Lin, J. Andrew Yeh

**Affiliations:** 1 Institute of NanoEngineering and Microsystems, National Tsing Hua University, No. 101, Section 2, Kuang-Fu Road, Hsinchu 30013, Taiwan; sujeet.nthu@gmail.com (S.K.R.); Arthur_Kao@epistar.com (K.-W.K.); ashishag07@gmail.com (A.A.); 2 Institute of Electronics Engineering, National Tsing Hua University, Hsinchu 30013, Taiwan; s104063528@m104.nthu.edu.tw; 3 Department of Physics, National Tsing Hua University, Hsinchu 30013, Taiwan; gwo@phys.nthu.edu.tw

**Keywords:** InN, selectivity, silicone oil, external filter, liver malfunction, exhaled-breath volatile organic compound (VOCs)

## Abstract

Ammonia is an essential biomarker for noninvasive diagnosis of liver malfunction. Therefore, selective detection of ammonia is essential for medical application. Here, we demonstrate a portable device to selectively detect sub-ppm ammonia gas. The presented gas sensor is composed of a Pt coating on top of an ultrathin Indium nitrite (InN) epilayer with a lower detection limit of 0.2 ppm, at operating temperature of 200 °C, and detection time of 1 min. The sensor connected with the external filter of nonpolar 500 CS silicone oil to diagnose liver malfunction. The absorption of 0.7 ppm acetone and 0.4 ppm ammonia gas in 10 cc silicone oil is 80% (0.56 ppm) and 21.11% (0.084 ppm), respectively, with a flow rate of 10 cc/min at 25 °C. The absorption of acetone gas is 6.66-fold higher as compared to ammonia gas. The percentage variation in response for 0.7 ppm ammonia and 0.7 ppm acetone with and without silicone oil on InN sensor is 17.5% and 4%, and 22.5%, and 14% respectively. Furthermore, the percentage variation in response for 0.7 ppm ammonia gas with silicone oil on InN sensor is 4.3-fold higher than that of 0.7 ppm acetone. The results show that the InN sensor is suitable for diagnosis of liver malfunction.

## 1. Introduction 

Significant research efforts have been dedicated to developing ammonia gas sensors for different applications, such as environmental monitoring and medical applications [1,2,3,4,5,6]. Ammonia gas has a large impact on the environment and human health. There are approximately 1840 volatile organic compounds (VOCs) generated by different parts of the body in healthy humans, and most VOCs are found in exhaled breath [3,7,8]. The concentration profile of exhaled-breath VOCs directly correlates with specific disease biomarkers such as ammonia for liver malfunction [9,10,11,12,13,14], acetone for diabetes [15,16,17,18,19], dimethyl sulfide (DMS) for liver malfunction [20,21], and isoprene for cholesterol [22]. Clinical data shows that the ammonia concentration in the breath is less than 0.278 part per million (ppm) for healthy people, while increases from 0.278 to 5 ppm for liver malfunction [13]. The dimethyl sulfide concentration in exhaled breath increases from 10 to 60 part per billion (ppb) for liver disease [20]. The acetone concentration in the breath varies from 0.3–0.9 ppm for healthy people while for diabetic patients it exceeds to 1.8 ppm [16,17,18,23]. The isoprene concentration in exhaled breath is 20–234 ppb [22], which is lower in the case of children [24]. The percentage of carbon dioxide (CO_2_) in the breath is about 4–5%. Therefore, the selective sensing of (< 100 ppb) ammonia gas remains a key challenge in medical diagnostics, combined with a complex matrix of exhaled-breath interferents to indicate liver malfunction. 

Recently, semiconductor gas sensors have been in high demand for the noninvasive detection of biomarkers because they have high sensitivity, provide a stable response and have low detection limits [25,26,27,28,29,30,31,32,33,34]. Much research has been focused on metal oxide semiconductor (MOS)-based ammonia gas sensor [35,36,37,38,39,40]. However, metal oxide semiconductor gas sensors have poor selectivity because a wide range of operation temperature leads to a wide range of sensitivity to the VOCs [41]. Owing to recent advances in epitaxial growth of InN crystalline films, high-quality ultrathin InN epilayers have been obtained. Ultrathin InN has exceptional electronic properties, such as a narrow band gap (0.6−0.7 eV), excellent electron transport characteristics (mobility > 1000 cm^2^/V·s), and high electron density (typically in excess of 1 × 10^18^ cm^−3^) for nominally undoped InN films [42]. In addition, the sheet carrier density linearly increases with thickness of the InN epilayer. Therefore, residual sheet charge is found to be $4.3\times 10^{13}$ ${\mathrm{cm}}^{-2}$ with an unusual electron accumulation layer within a few nanometers of the InN surface [43,44,45,46,47]. Therefore, an ultrathin InN epilayer has high sensitivity for ammonia gas in exhaled breath. However, the InN gas sensor has a lack of selectivity. To improve the selectivity, there are various techniques available such as synthesizing unique, highly sensitive materials for the target gas but with a negligible cross sensitivity for interfering gases, as well as temperature modulation techniques [48,49,50,51,52,53,54] and gas sensor arrays. [8,55,56,57,58] using filters combined with a sensing layer can reduce the sensitivity for interfering gases and enhance the selectivity of the gas sensor. However, combining a sensing layer with filters may lead to decreased sensitivity of the gas sensor and the detection limit. Previously, our group also reported an InN-based gas sensor and its numerous applications in medical diagnosis [59,60,61]. 

**Figure 1:**
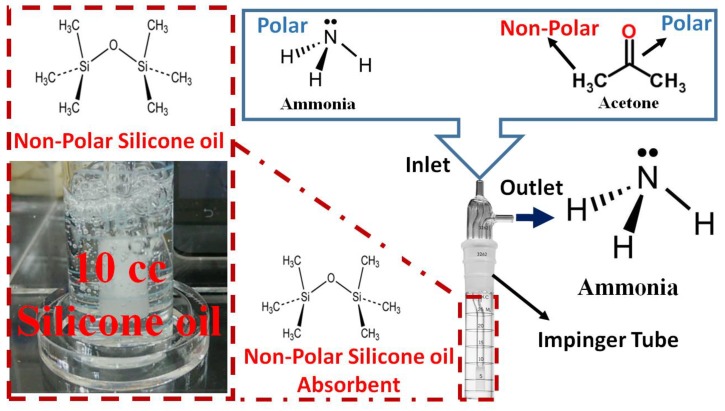
Absorption of ammonia and acetone gas in 10 cc silicone oil into the impinger tube.

In this study, we report chemically inert and nonpolar 500 centistokes (CS) O-ring silicone oil as an external filter to enhance the selectivity of the InN gas sensor. The filter is separate from the ultrathin InN epilayer and it is used to filter out the major interferents—acetone gas—in exhaled breath, thus enhancing the selectivity of the InN gas sensor for ammonia gas in liver malfunction applications. Silicone oil is a nonpolar solvent that can absorb acetone gas [62]. Acetone gas possesses two different kinds of chemical groups, a carbon monoxide (–CO) polar group and a methyl (–CH_3_) nonpolar group. Therefore, acetone has polar and nonpolar characteristics. In addition, ammonia is a strongly polar gas that forms hydrogen bonds. Therefore, when the ammonia and acetone gas pass through 10 cubic centimeter (cc) silicone oil into the impinger tube, the major interferent acetone gas is absorbed into the silicone oil, while the target ammonia gas has negligible absorption into the silicone oil, as shown in Figure 1. Furthermore, the polarity of ammonia is not matched with the polarity of silicone oil. However, polarity of acetone gas is matched by the polarity of silicone oil. Therefore, acetone has much greater absorption than ammonia gas in 10 cc silicone oil. There are other interferents in the exhaled breath beside acetone gas, such as isoprene, CO_2,_ and dimethyl sulfide (DMS). The concentration of these VOCs is much smaller than the major interferent acetone gas. In addition, nonpolar isoprene will be absorbed into the silicone oil. The nonpolar carbon dioxide (CO_2_) is also absorbed into the silicone oil [63]. Dimethyl sulfide has a negligible response on the InN sensor, because the concentration of DMS in the breath is very low. Therefore, these interferents have a negligible effect on the target ammonia detected by the InN sensor. Thus, selectivity for ammonia gas is improved. Furthermore, the absorption of acetone gas also depends on the gas flow rate at which the acetone gas passes through the silicone oil. In addition, exhaled-breath flow rate for healthy people changes from 5–10 L/min [64]. However, the concentration of exhaled-breath VOCs is inversely proportional to the breath flow rate 50–400 cc/min [65]. Furthermore, low gas flow rates lead to increased absorption because the number of the smaller-sized bubbles in the silicone oil increases, leading to increased contact time between the gas bubbles and the silicone oil. When the gas flow rate is high, fewer larger-sized bubbles are created. Therefore, the contact time of the gas bubbles with the silicone oil decreases, leading to decreased absorption [66,67]. The amount of acetone and ammonia gas absorbed inside the silicone oil is given by Equation (1).

(1) $$\frac{m\left(t\right)}{\sqrt{t}}\;=\;2c_{s}\sqrt{\frac{D}{\pi }}\left(1\;+\;\frac{kt}{4}\right)$$

where t is the contact time of the gas bubbles with the silicone oil, k is the first-order reaction velocity constant, m(t) is the amount of gas absorbed into the liquid, D is the diffusion coefficient, and $C_{s}$ is the gas-liquid interface concentration. The value $\left[C_{s}\sqrt{D}\right]\times 10^{-5}kgm^{-2}s^{-\frac{1}{2}}$ = 0.79, 0.73, or 0.7 and the reaction velocity constant [*k*] $s^{-1}$ = 0.56, 0.8, or 1.4 and the temperatures of 293 K, 298 K, or 303 K, respectively [66].

## 2. Method and Setup

### 2.1. Sampling Process

A gas chamber unit is used to prepare the 0.7 ppm acetone gas sample in nitrogen background. The gas chamber unit consists of a gas supply unit, a chamber unit, a temperature control unit, and a current measurement unit. The gas supply unit is comprised of a one-way valve, a mass flow control (MFC) unit and a gas mixture unit. 

A vacuum pump connected to a gas chamber is used to clean the chamber. Before the preparation of 0.7 ppm acetone gas sample, the chamber is cleaned for 5 min then a 1-Liter Bag1 is connected to the gas chamber for 20 min, as shown in Figure S1. After collection of the gas sample, the bag is disconnected from the chamber. When the same method is used for the preparation of sub-ppm ammonia gas sample, collected into the tedlar bag using MFC unit, the analytical instrument shows an unstable response, which makes the accurate quantification of the sub-ppm ammonia gas difficult using the analytical instrument. Hence, to avert this, the sampling method for ammonia gas sample preparation is changed to a flow meter from an MFC unit. 

Therefore, to sample 0.4 ppm ammonia gas, a 10-ppm standard ammonia cylinder is connected to a flow meter with a maximum flow rate of 100 cc/min. In addition, a nitrogen cylinder is connected to the air flow meter with a maximum flow rate of 5 liters/min. To sample 0.4 ppm ammonia gas, a 10-Liter Tedlar Bag1 is connected to the 10-ppm ammonia cylinder with a gas flow rate of 100 cc/min for 4 min, as shown in Figure S2a then 400 cc of ammonia gas was diluted with 10 liters of N2 gas with a flow rate of 5 liters/min for 2 min, as shown in Figure S2b.

### 2.2. Absorption Setup of Acetone

**Figure 2:**
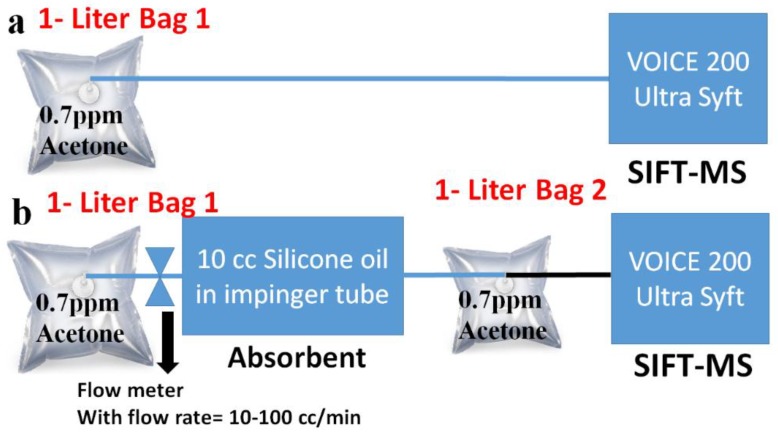
(**a**) To quantify the concentration of the 0.7 ppm standard acetone gas by SIFT-MS. (**b**) Absorption measurement of 0.7 ppm acetone gas through 10 cc silicone oil by using SIFT-MS.

The absorption of 0.7 ppm acetone gas in 10 cc silicone oil is measured by using selected ion flow tube mass spectrometry SIFT-MS (VOICE 200 Ultra Syft, Syft Technologies Ltd. Christchurch, New Zealand). The 1-Liter Bag1 is connected to the inlet of the SIFT-MS to measure the concentration of 0.7 ppm standard acetone gas, as shown in Figure 2a. Furthermore, the 1-Liter Bag1 is connected to the inlet of the impinger tube through the flow meter, and the 1-Liter Bag2 is connected to the outlet of the impinger tube. Thereafter, 0.7 ppm acetone gas flows through the 10 cc silicone oil inside the impinger tube at a controlled flow rate, such as 10 cc/min or 20 cc/min. After absorption in 10 cc silicone oil, the acetone gas is collected in 1-Liter Bag 2 as shown in Figure 2b. Furthermore, the 1-Liter Bag 2 is connected to the inlet of the SIFT-MS to measure the absorption of 0.7 ppm acetone gas in 10 cc silicone oil.

### 2.3. Absorption Setup of Ammonia

**Figure 3:**
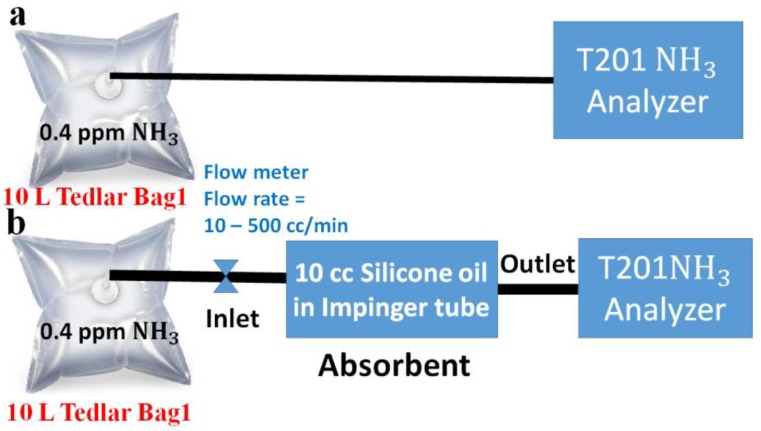
(**a**) The concentration measurement of the 0.4 ppm standard ammonia gas by using T201 NH_3_ Analyzer. (**b**) Absorption measurement of 0.4 ppm ammonia gas into 10 cc silicone oil by T201 NH_3_ Analyzer.

The absorption of ammonia gas in 10 cc silicone oil is measured by using T201 NH_3_ Analyzer. The 10-Liter Tedlar Bag1 is connected to the inlet of the T201 chemiluminescence-based NH_3_ analyzer (Teledyne-API) to measure the concentration of 0.4 ppm standard ammonia gas, as shown in Figure 3a. Furthermore, the 10-Liter Tedlar Bag1 is connected to the inlet of the impinger tube, and the outlet of the impinger tube is connected to the inlet of the T201 NH_3_ Analyzer to measure the absorption of 0.4 ppm ammonia gas in 10 cc silicone oil with gas flow rates of 10 cc/min, 20 cc/min, 500 cc/min, as shown in Figure 3b.

### 2.4. Sensitivity of InN Gas Sensor

The gas sensor sensitivity is defined in terms of the current variation ratio, the relative current change upon exposure to the analyte gas to the current in the ambient gas, which can be expressed as per Equation (2).

(2) $$\mathrm{Sensitivity}\left(\frac{\Delta I}{I_{o}}\right)=\;\frac{I_{\mathrm{Analyte}\;\mathrm{gas}}-I_{\mathrm{Ambient}\;\mathrm{gas}}}{I_{\mathrm{Ambient}\;\mathrm{gas}}}$$

Current in presence of the analyte gas given by Equation (3)

(3) $$I_{\mathrm{Analyte}\;\mathrm{gas}}=µ\left(n_{o}+\Delta n_{\mathrm{Analyte}\;\mathrm{gas}}\right){\mathrm{eEA}}_{\;}$$

Current in presence of the ambient gas given by Equation (4)

(4) $$I_{\mathrm{Ambient}\;\mathrm{gas}}={µn}_{o}{\mathrm{eEA}}_{\;}$$

Gas sensor sensitivity changes with electron density given by Equation (5)

(5) $$\mathrm{Sensitivity}\left(\frac{\Delta I}{I_{o}}\right)=\frac{\Delta n_{\mathrm{Analyte}\;\mathrm{gas}}}{n_{o}}$$

The name of the symbols used in Equation (2) to Equation (5) are given in Table 1.

In presence of the analyte gas, the sensitivity of gas sensor varies linearly with the number of the electron density.

## 3. Ultrathin InN Gas Sensor 

### 3.1. Fabrication Process

The flow process of the single crystalline InN film on sapphire substrates is briefly described as follows. The plasma-assisted molecular beam epitaxy is used to accomplish the process with a radio frequency generated by nitrogen plasma source. The indium source is produced by thermal evaporation of the pure source materials. During deposition, the base pressure of the MBE system is kept at an ultrahigh vacuum of 1 × 10^−9^ Torr. The nitrogen plasma has high reactivity with evaporated indium atom flux on the substrate, allowing InN growth at low temperature. Two-inch sapphire Al_2_O_3_ (0001) wafers are used as starting substrates. A buffer layer, aluminum nitrite (AlN) 500 nm, is deposited prior to the growth of InN to minimize the large lattice mismatch between InN (0001) and Al_2_O_3_ (0001). For the growth of InN, a low-temperature buffer layer AlN is grown at 350 °C, followed by high-temperature InN growth at 520 °C. The heater in the serpentine structure is composed of an aluminum film with a thickness of 200 nm on the glass substrate. The fabrication processes of the heater and InN epilayer are shown in Figure S3a,b, respectively.

### 3.2. Gas Sensor Device

The gas sensor device is contained in an ultrathin (~10 nm) InN epilayer with a pair of electrodes composed of an Ti/Al/Au (50 nm/200 nm/50 nm) composite grown on the sapphire substrate along with an AlN buffer layer. The deposition of the electrodes on the InN epilayer is conducted in an electron-beam evaporation system. The Ti/Al/Au composite electrode forms an ohmic contact with InN epilayer, due to low resistivity between electrodes and InN [68,69]_._ Which is used to measure the current of the InN conductive channel between the electrodes. A very thin platinum (Pt) film (~10 nm) is deposited between the two electrodes on the InN surface. Because platinum has been used in many catalytic applications than other catalytic metals such as gold and silver because Pt has the capability to interact with poisons such as sulfur compounds that are limited to the metal surface. Pt has a high melting point; Pt has efficiently been recycled [70]. On the other hand, the cost of the Pt is very high, due to the limited supply of the Pt. In addition, the major issue with Pt is one of a singular geology [71]. The advantage and disadvantage of the Pt shown in Table 2.

**Figure 4:**
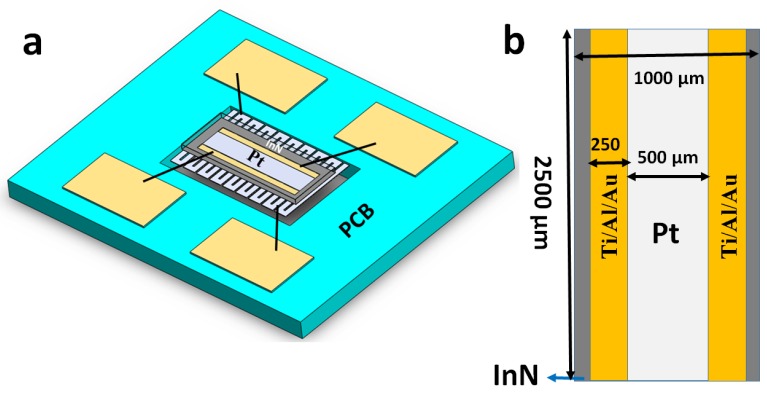
(**a**) The InN gas sensor device in PCB (**b**) The electrode Ti/Al/Au connected with InN epilayer.

The deposition of Pt film is conducted in an electron-beam evaporation system. Furthermore, for higher operating temperatures, an aluminum heater is included, which is connected to the back side of the ultrathin InN epilayer by thermally conductive silicone adhesive that allows heat conduction. In addition, heater along with platinum coating on the InN epilayer is inserted into the hole in printed circuit board (PCB) substrate along with four bonding pads in Figure 4a. The dimension of the InN sensing layer along with platinum layer between two electrodes Ti/Al/Au in Figure 4b. Due to the Pt layer on the InN epilayer, sensitivity and response time are improved. When acetone and ammonia gas is exposed on Pt coating on the InN sensor. They are adsorbed on the adsorption sites and further dissociated into hydrogen atom at platinum catalytic film [72,73,74,75,76]. In addition, part of the dissociated hydrogen atom diffuses and are trapped at the interface of Pt–InN epilayer to form the dipole layer at the interface [77]. Therefore, dipole layer creates an additional electric field at the interface, leads to modulate the conductivity of the InN epilayer. Thus, the sensitivity of the InN sensor will be improved. The operating temperature of the heater can be enhanced up to 200 °C. The temperature of the InN sensing layer increases as the heater temperature increases. Therefore, the gas sensing response is enhanced at 200 °C.

#### Sensing Mechanism

**Figure 5:**
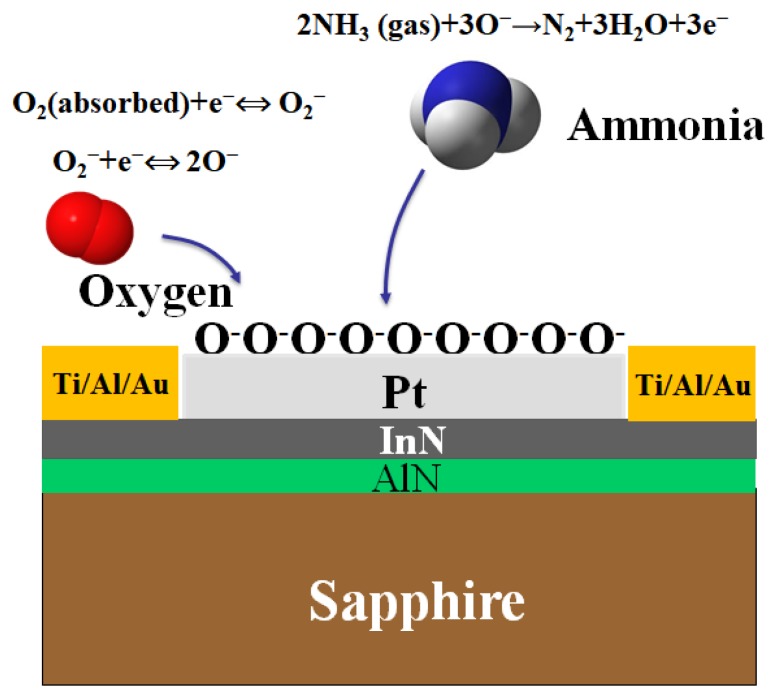
Sensing mechanism of ammonia gas on the Pt coated InN gas sensor.

In air background, and at operating temperature of 200 °C, oxygen molecules are adsorbed onto the surface of the InN epilayer to form the $O_{2}^{-}$ ions by attracting the electron from the conduction band of the InN epilayer. Furthermore, when ammonia gas reacts with oxygen ion on the InN surface, ammonia transfer their lone pair electron into the conduction band of the InN epilayer. Therefore, conductivity of the InN epilayer increases. Consequently, InN gas sensor has high sensitivity for the ammonia gas in Figure 5. 

### 3.3. Function of Valves and Pump System

**Figure 6:**
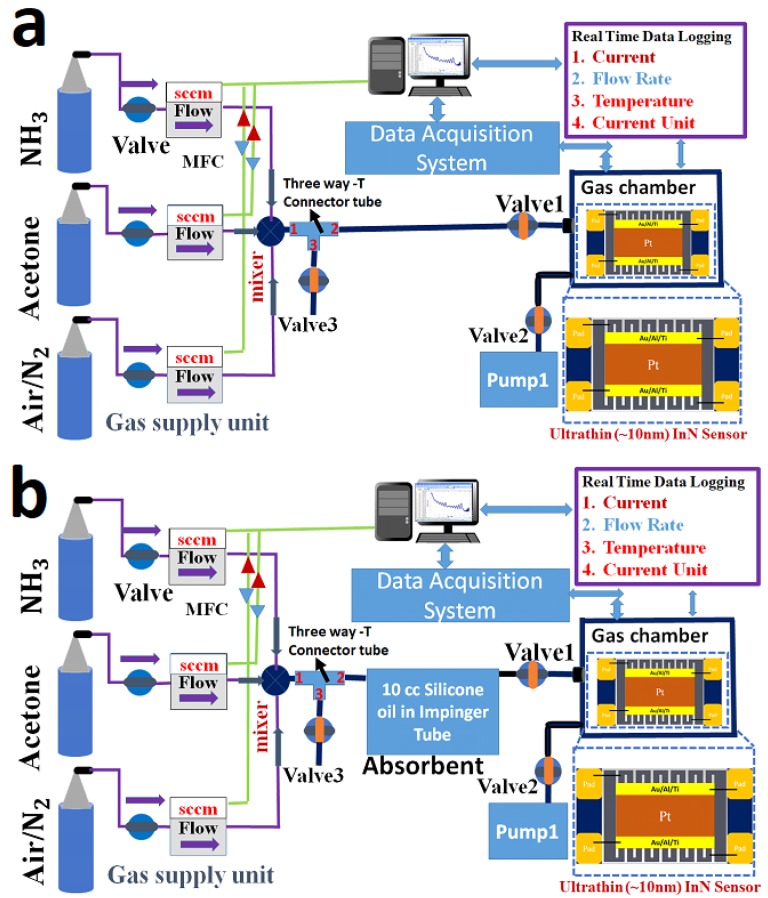
(**a**) Portable gas sensor system comprised three valves and pump system. (**b**) Portable gas sensor system connected with gas supply unit through the impinger tube consists of the 10 cc silicone oil.

A portable gas sensor system is used to measure the sub-ppm ammonia and acetone gas responses with and without silicone oil. The volume of the portable gas chamber is 9.8 mL. A valve and pump system are used to reduce the detection time of the portable gas sensor to approximately 1 min for real-time applications in exhaled-breath analysis for liver malfunction applications. Here, three electromagnetic valves and one pump are used, and they are named as Valve1, Valve2, Valve3, and Pump1, respectively. In this system, a three-way T-connector tube is used. Inlet port 1 of the T-connector tube is connected to the gas mixing unit, port 2 of the connector is connected to the inlet of Valve1, and the outlet of Valve1 is connected to the inlet of the portable gas chamber unit. In the same way, port 3 of the connector is connected to the inlet of Valve3, and the outlet of Valve3 is open as a bypass to release the gas. The inlet of Valve2 is connected to the exhaust port of the portable gas chamber unit, and the outlet of Valve2 is connected to the inlet of Pump1 to improve the response and recovery times, as shown in Figure 6a. The valve and pump system operation is controlled by an RS-232 circuit board. To measure the selectivity of the ultrathin InN portable gas sensor for measuring sub-ppm ammonia gas over acetone gas, 10 cc silicone oil is used in the impinger tube as an external filter to filter out the undesired acetone gas. Therefore, the gas mixing unit is connected to the impinger tube through the three-way T-connector tube with the three valve and pump system. Inlet port 1 of the T-connector tube is connected to the gas mixing unit, port 2 of the connector is connected to the inlet of the impinger tube, the outlet of the impinger tube is connected to the inlet of Valve1, and the outlet of Valve1 is connected to the inlet of the portable gas chamber unit. In the same way, port 3 of the T-connector tube is connected to the inlet of Valve3, and the outlet of Valve3 is open as a bypass to release the gas. The inlet of Valve2 is connected to the exhaust port of the portable gas chamber unit, and the outlet of Valve2 is connected to the inlet of Pump1 to improve the response and recovery times, as shown in Figure 6b.

## 4. Results and Discussion

The absorption of 0.7 ppm acetone and 0.4 ppm ammonia gas is measured through 10 cc silicone oil in an impinger tube by using the (200 VOICE Ultra Syft) SIFT-MS system with a 10 ppb resolution and a T201 chemiluminescence-based NH_3_ Analyzer with a detection limit of (1 ppb), respectively. The absorption setup of acetone and ammonia gas is discussed in Section 2.2 and Section 2.3, respectively. 

**Figure 7:**
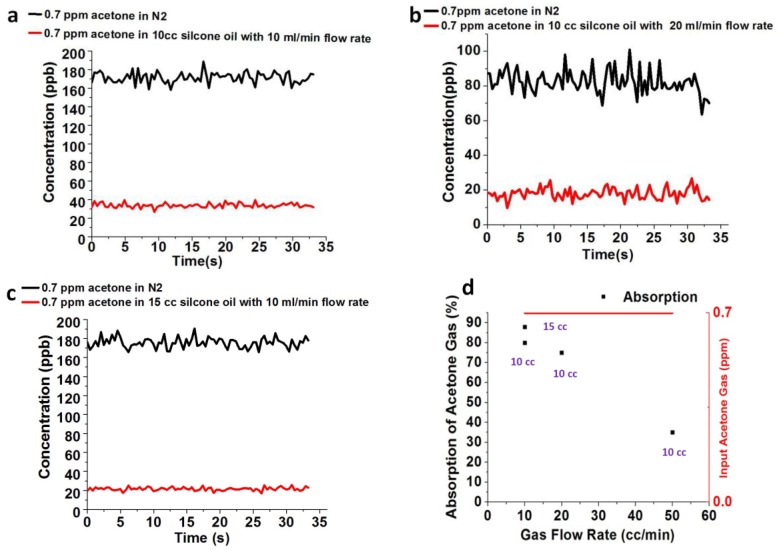
(**a**) Absorption measurement of 0.7 ppm acetone in 10 cc silicone oil with gas flow rate 10 cc/min (**b**) Absorption measurement of 0.7 ppm acetone in 10 cc silicone oil with gas flow rate 20 cc/min (**c**) Absorption measurement of 0.7 ppm acetone in 15 cc silicone oil with gas flow rate 10 cc/min. (**d**) Absorption of 0.7 ppm acetone gas in 10 cc and 15 cc silicone oil at the flow rate of 10 cc/min, 20 cc/min and 50 cc/min.

The concentration of 0.7 ppm acetone gas in the 1-Liter bag1 is measured by SIFT-MS, also, the absorption of 0.7 ppm acetone gas through 10 cc silicone oil is measured at different flow rate by SIFT-MS. The absorption response of 0.7 ppm acetone gas is higher at a flow rate 10 cc/min (Figure 7a) in comparison to the absorption at a flow rate 20 cc/min (Figure 7b). As the acetone gas flow rate increases, the absorption of 0.7 ppm acetone gas into 10 cc silicone oil decreases. Furthermore, the absorption of 0.7 ppm acetone gas in 15 cc silicone oil with a gas flow rate 10 cc/min is measured, as shown in Figure 7c. When the amount of silicone oil increases, the absorption of acetone gas also increases at a flow rate of 10 cc/min. 

The maximum absorption of acetone gas reaches approximately 88% in 15 cc silicone oil and 80% in 10 cc silicone oil at a gas flow rate 10 cc/min, respectively. The absorption of acetone gas is reduced to 75% and 40% at gas flow rate of 20 cc/min and 50 cc/min, respectively, as shown in Figure 7d.

**Figure 8:**
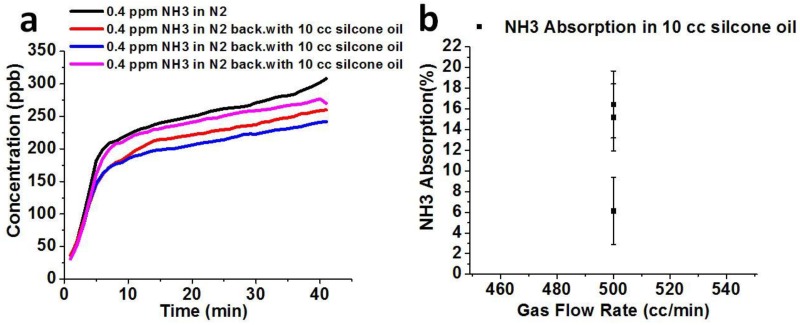

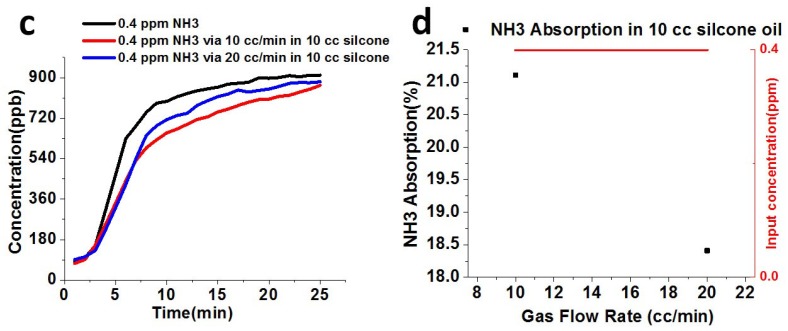
(**a**) Concentration of ammonia detected in 10 cc silicone oil and without silicone oil at 500 cc/min (**b**) Absorption of ammonia gas in 10 cc silicone oil at 500 cc/min. (**c**) The concentration of ammonia detected with 10 cc silicone oil and without silicone oil at 10 cc/min and 20 cc/min (**d**) Absorption of ammonia gas in 10 cc silicone oil at 10 cc/min and at 20 cc/min.

The responses of 0.4 ppm ammonia with and without 10 cc silicone oil are measured by using the T201 NH_3_ Analyzer as shown in Figure 8a. Approximately 6.13 to 16.42% ammonia gas is absorbed in 10 cc silicone oil at a flow rate of 500 cc/min, as shown in Figure 8b. As the flow rate of 0.4 ppm ammonia gas through 10 cc silicone oil is decreased to 20 cc/min and 10 cc/min as shown in Figure 8c, its absorption increases to 18.4% and 21.11%, respectively, as shown in Figure 8d. This result reflects a negligible amount of ammonia absorption in 10 cc silicone oil as compared to acetone gas.

**Figure 9:**
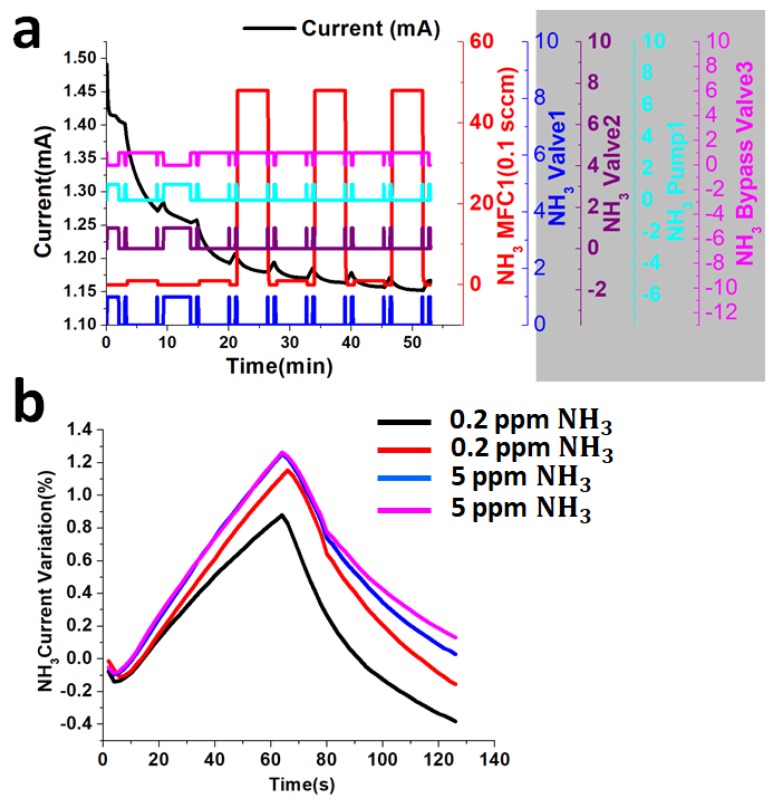
(**a**) The response of 0.2 ppm and 5 ppm ammonia gas on ultrathin InN gas sensor (**b**) Current variation response for 0.2 ppm and 5 ppm ammonia gas.

To measure the current response on the Pt-coating on top of InN gas sensor, the valve and pump system is used for improving the detection time. The detailed operation of the valve and pump system is discussed in Section 3.3. When sub-ppm ammonia gas concentrations, such as 0.2 ppm and 5 ppm, are exposed on the Pt coating on top of the ultrathin InN epilayer at 200 °C with a gas flow rate of 500 sccm as shown in Figure 9a. The current variation response for the 0.2 ppm and 5 ppm ammonia is 0.8% and 1.3%, respectively, with a detection time of 1 min, as shown in Figure 9b.

**Figure 10:**
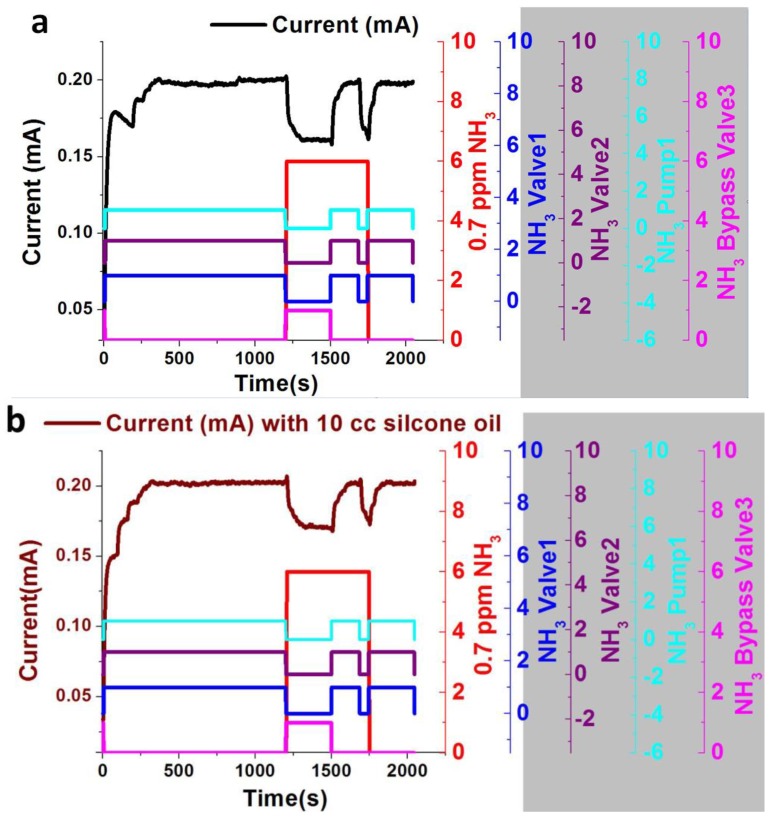

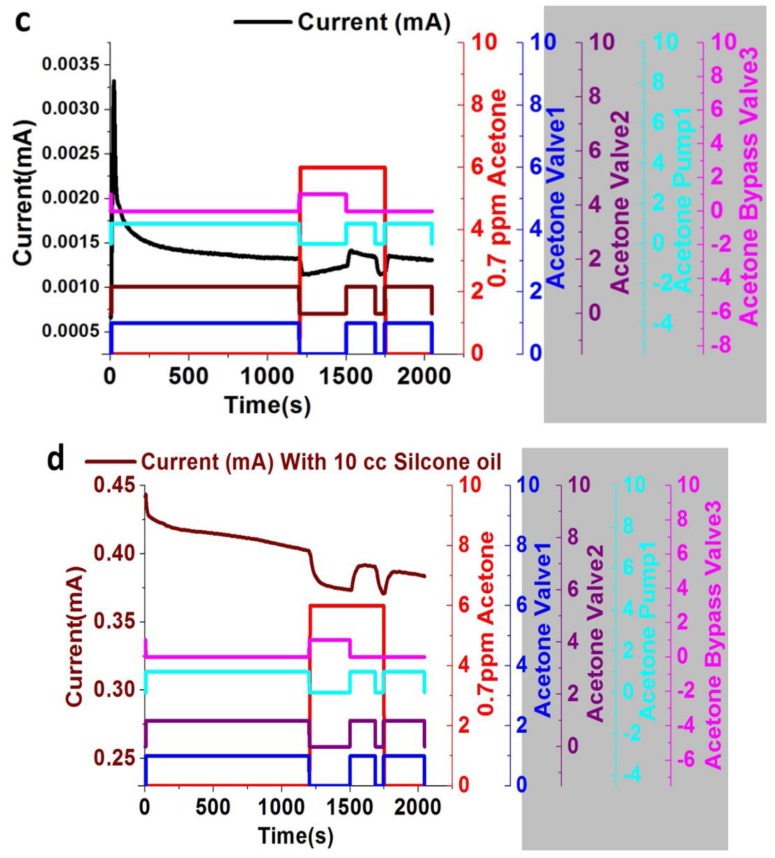
(**a**) The current response of 0.7 ppm ammonia gas. (**b**) The current response of 0.7 ppm ammonia gas with 10 cc silicone oil. (**c**) The current response of 0.7 ppm acetone gas. (**d**) The current response of 0.7 ppm acetone gas with 10 cc silicone oil.

To enhance the selectivity of the ultrathin InN gas sensor, nonpolar silicone oil is used as an absorbent to absorb interfering acetone gas, consequently enhancing the signal-to-noise ratio of the target ammonia gas. Furthermore, 0.7 ppm ammonia gas and 0.7 ppm acetone gas are used to measure the variation in the current response on the ultrathin InN gas sensor with and without 10 cc silicone oil. The portable gas chamber system is used to expose 0.7 ppm ammonia gas and 0.7 ppm acetone gas on the ultrathin InN epilayer as shown in Figure 6a. In addition, 0.7 ppm ammonia gas and 0.7 ppm acetone gas flow through the impinger tube containing 10 cc silicone oil on the ultrathin InN epilayer via the portable gas chamber system as shown in Figure 6b. The current responses for 0.7 ppm ammonia without and with silicone oil are shown in Figure 10a,b, respectively, while the current responses for 0.7 ppm acetone without and with silicone oil are shown in Figure 10c,d, respectively.

**Figure 11:**
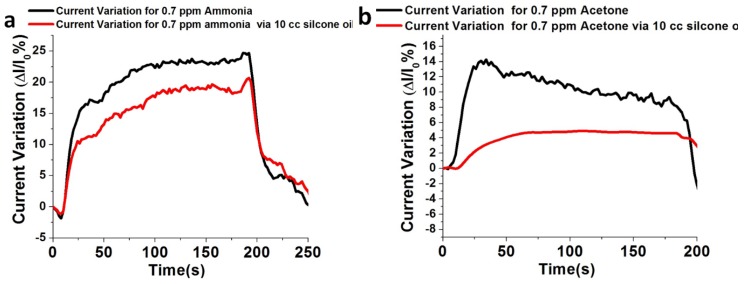
(**a**) Current variation response 0.7 ppm ammonia gas with 10 cc silicone oil and without silicone oil. (**b**) Current variation response 0.7 ppm acetone gas with 10 cc silicone oil and without silicone oil.

The current variation response of 0.7 ppm ammonia gas with 10 cc silicone oil $\left(\frac{\Delta I}{I_{o}}\%=17.5\right)$ and without silicone oil $\left(\frac{\Delta I}{I_{o}}\%=22.5\right)$, and the current variation response of 0.7 ppm acetone gas with 10 cc silicone oil $\left(\frac{\Delta I}{I_{o}}\%=4\right)$ and without silicone oil $\left(\frac{\Delta I}{I_{o}}\%=14\right)$ are shown in Figure 11a,b, respectively.

**Figure 12:**
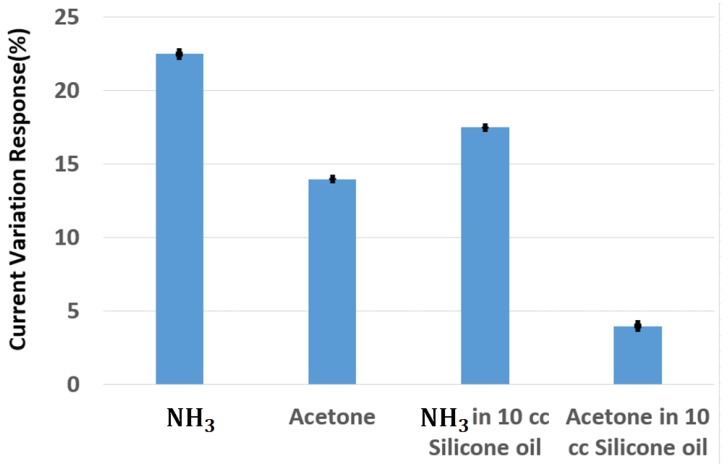
The current variation response for 0.7 ppm ammonia, 0.7 ppm acetone with and without 10 cc silicone oil on the InN gas sensor.

Therefore, the current variation response for 0.7 ppm ammonia gas in 10 cc silicone oil is 4.3-fold higher than the current variation response for 0.7 ppm acetone at 200 °C as shown in Figure 12. In addition, the current variation for the 0.7 ppm ammonia without silicone oil is only 1.6-fold higher than the current variation response for the 0.7 ppm acetone on the InN gas sensor. 

## 5. Detection Limit of Portable System 

The detection limit of potable gas sensor system for ammonia and acetone gas with the external filter of silicone oil in Table 3.

## 6. Conclusions

Silicone oil is an effective absorbent for improving the selectivity of ultrathin InN gas sensors to detect ammonia gas for liver malfunction applications. In an exhaled breath, ammonia gas is a biomarker for liver malfunction, and the major interfering gas is acetone gas. When acetone and ammonia gas are passed through silicone oil, the absorption of 0.7 ppm acetone gas is 6.66-fold higher than the absorption of 0.4 ppm ammonia gas at a flow rate of 10 cc/min, because acetone is nonpolar, whereas ammonia is polar. After measuring the absorption through the silicone oil, a chip test was performed for 0.7 ppm acetone gas and 0.7 ppm ammonia gas through 10 cc silicone oil at a gas flow rate of 500 sccm on the ultrathin InN gas sensor at 200 °C. The current variation response for 0.7 ppm ammonia gas in 10 cc silicone oil is 4.3-fold higher than the current variation response for 0.7 ppm acetone. The lower detection limit of the portable InN gas sensor system with external filter of silicone oil for ammonia and acetone gas is as low as 0.242 ppm and 0.36 ppm respectively with a detection time of 1 min at 200 °C, which allows the system to be used for the noninvasive and selective detection of sub-ppm ammonia gas in exhaled-breath VOCs for medical applications in diagnosing liver malfunction.

## Figures and Tables

**Table 1:** The name of symbols used in Equations (2)–(5).

Symbols	Name
µ	Mobility
$n_{o}$	Electron density in ambient gas
e	Electronics charge
E	Electric field
A	Cross sectional area
$\Delta n_{\mathrm{Analyte}\;\mathrm{gas}}$	Electron density in presence of analyte gas

**Table 2:** The advantage and disadvantage of the Pt catalyst.

Pt Catalyst	Advantage [70]	Disadvantage [71]
	High melting point	Cost is very high
	Has ability to interact with poisons and Sulfur compound	Limited supply
	Efficiently recycled	Geological singularity

**Table 3:** Detection limit of portable gas sensor system with external filter of silicone oil.

Parameter	Before Absorption	After Absorption	Silicone Oil	Flow Rate
Detection limit NH_3_	0.2 ppm	0.242 ppm	10 cc	10 cc/min
Detection limit of Acetone	0.2 ppm	0.36 ppm	10 cc	10 cc/min

## Supplementary Materials

The following are available online at http://www.mdpi.com/1424-8220/18/11/3887/s1, Figure S1: Acetone gas sampling in 1-liter bag1 by using gas chamber system, Figure S2: (a) Sampling of 0.4 ppm ammonia gas in 10-liter of Tedlar bag1 (b) N2 dilution system, Figure S3: (a) Fabrication process of heater (b) Fabrication process of InN Epilayer.
