# Supplementary material for: Indium Nitrite (InN)-Based Ultrasensitive and Selective Ammonia Sensor Using an External Silicone Oil Filter for Medical Application
Source: Sensors (Basel). 2018 Nov 11;18(11):3887. doi: 10.3390/s18113887 (PMC6263719; doi:10.3390/s18113887)
Supplement: Supplementary file 1 [file sensors-18-03887-s001.pdf]

Indium nitrite (InN)-based ultrasensitive and selective ammonia sensor using an external silicone oil filter for medical application

Sujeet Kumar Rai[1], Kun-Wei Kao[1], Shanjgr Gwo,[3] Ashish Agarwal,[1] Wei Da Lin[2], J. Andrew Yeh[1,2*]

[1]Institute of Nano engineering and Microsystems, National Tsing Hua University, No. 101, Section 2, Kuang-Fu Road, Hsinchu 30013, Taiwan.

[2] Institute of Electronics Engineering, National Tsing Hua University, Hsinchu 30013, Taiwan.

[3]Department of Physics, National Tsing Hua University, Hsinchu 30013, Taiwan.

*Correspondence and requests for materials should be addressed to J.A.Y.

(E-mail: jayeh@mx.nthu.edu.tw)

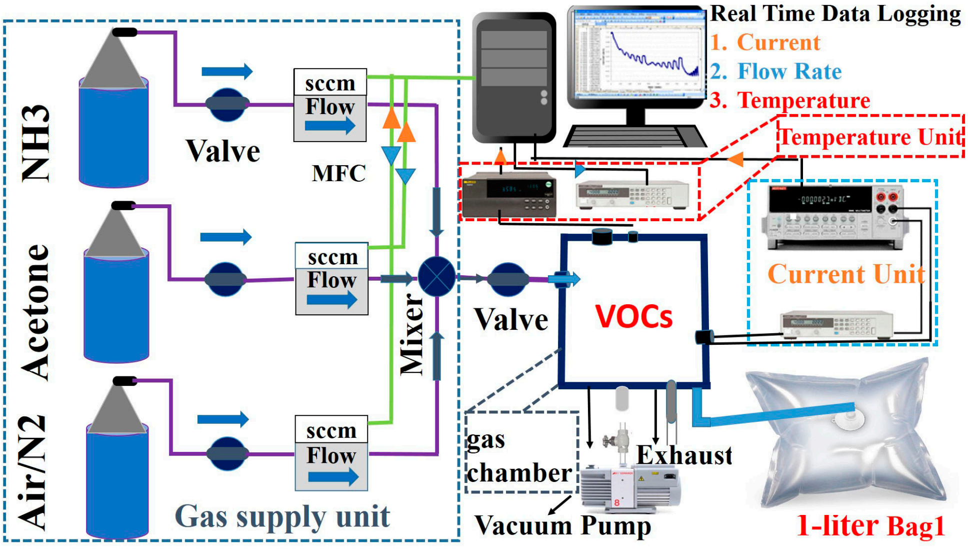

Figure S1. Acetone gas sampling in 1-liter bag1 by using gas chamber system.

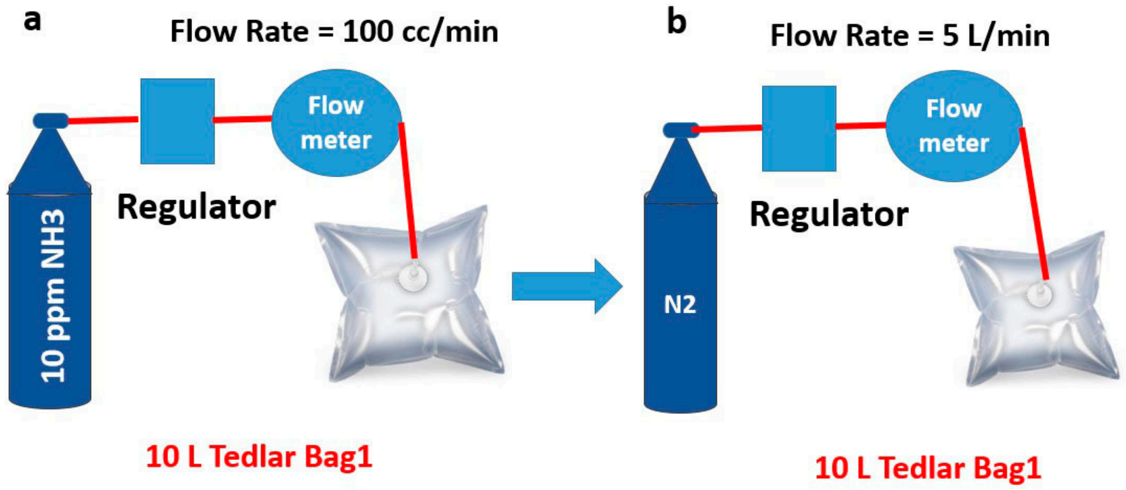

Figure S2. (a) Sampling of 0.4 ppm ammonia gas in 10-liter of Tedlar bag1 (b) N2 dilution system.

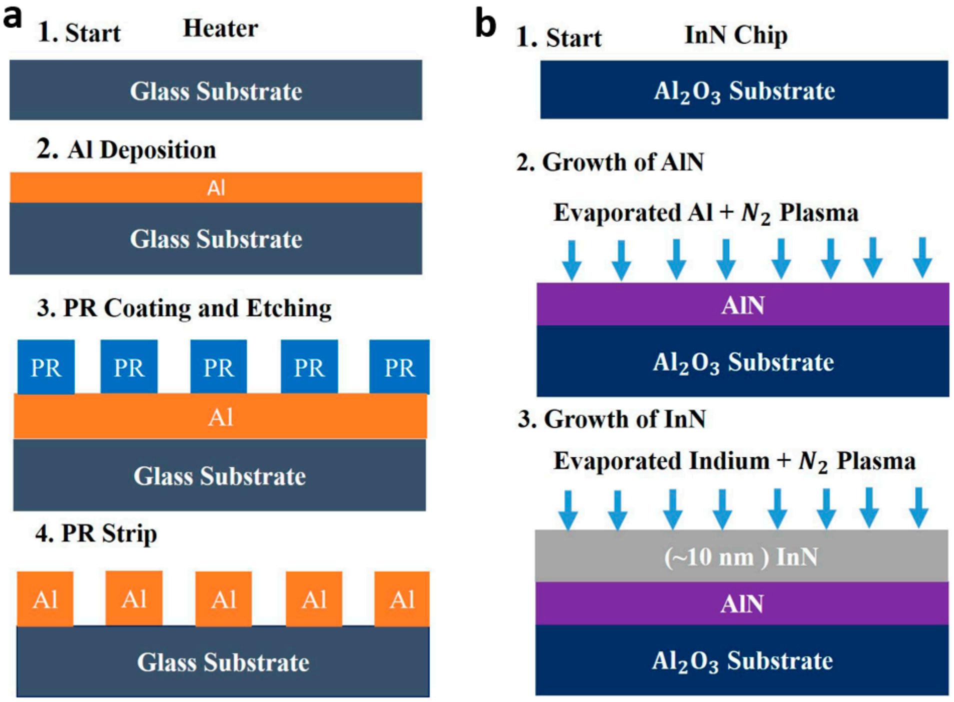

Figure S3. (a) Fabrication process of heater (b) Fabrication process of InN Epilayer.
